# System Mapping of Farm-to-School Partnerships to Enhance Student Access to Healthy, Local Foods

**DOI:** 10.3390/ijerph22091342

**Published:** 2025-08-27

**Authors:** Melissa Guillen, Katherine E. Soule

**Affiliations:** 1 Cooperative Extension in San Luis Obispo, Santa Barbara, and Ventura Counties, University of California, Santa Barbara, CA 93110, USA; mpguillen@ucanr.edu; 2 Department of Nutrition, University of California, Davis, CA 95616, USA

**Keywords:** farm-to-school, student nutrition, nutrition behaviors

## Abstract

Farm-to-school (F2S) partnerships connect schools with local producers to enhance student access to healthy, local foods and support regional food systems. Despite widespread implementation, few studies have examined the system-level structures that facilitate effective and sustainable F2S efforts across diverse community contexts. This study utilized a mixed-methods system mapping approach to analyze four F2S systems on California’s Central Coast. Data sources included public data sources, in-person site observations, and local expert resources. The researchers hypothesized that successful F2S systems would share core features that support implementation and long-term sustainability, including aligned values among stakeholders, the presence of identified champions, and multi-directional pathways for food procurement and communication. They constructed system maps and compared them in order to identify both common structural features and context-specific adaptations. Findings support the hypothesis and highlight the critical role of community-based organizations and distributors and/or aggregators in brokering relationships, coordinating resources, and reducing administrative burdens. Institutionalization through wellness policy integration and district-level support further distinguished higher-functioning systems. Identified barriers included inequities in procurement infrastructure, limited funder engagement, and uneven access to local food sources. System mapping offers a valuable tool to understand, strengthen, and scale equitable F2S implementation.

## 1. Introduction

Farm-to-school (F2S) initiatives aim to increase student access to locally grown foods through school meal programs. These initiatives aim to expand student access to healthy, locally sourced foods while offering experiential learning opportunities, ultimately fostering food literacy, strengthening local economies, and supporting communities [1]. Existing research on F2S initiatives tends to focus on several key areas: (1) student health and behavioral outcomes, particularly improvements in dietary intake, nutrition knowledge, self-efficacy, and food-related attitudes and behaviors [2,3,4,5,6,7,8,9,10,11,12,13,14]; (2) barriers to implementation and sustainability, including limited staff capacity, inadequate training, funding constraints, and procurement challenges [15,16]; and (3) impacts on participating farmers and school food service staff, such as expanding markets, economic benefits, and changes in procurement practices [17,18,19].

Logistical considerations can significantly impact the effectiveness of F2S initiatives. Distributors and aggregators, while often grouped together, serve distinct functions in the supply chain. Distributors primarily deliver products to end users, while aggregators consolidate products from multiple producers to streamline sales and distribution. In regional food systems, farmers or intermediary organizations may fulfill both functions, and in some cases may perform both roles simultaneously [20]. The use of distributors, aggregators, and centralized receiving hubs can simplify procurement for school food service directors by consolidating ordering and deliveries, though these efficiencies may result in increased transportation and handling fees [17], while direct purchasing from farmers can reduce costs and increase flexibility but also increase administrative burdens. Schools may also utilize the Fresh Produce Program to access local foods through existing commodity distribution networks in exchange for a small commission, which can simplify ordering, consolidate delivery, and offer competitive pricing for schools while helping to offset administrative costs for the federal government [18]. Successful F2S initiatives require individuals with the knowledge, relationships, and time to navigate these complex logistical systems. The presence of a committed ‘champion’ who unites stakeholders is frequently cited as a key factor for program success, and these individuals often play a central role in coordinating stakeholders, securing institutional support, and sustaining momentum in the face of logistical or bureaucratic challenges [15,16,17,18]. In the United States, recent national and state investments have expanded F2S initiatives, including USD 86.8 million for 375 California projects by the Department of Food and Agriculture between 2021 and 2024 [21]. Considering the significant investment in F2S initiatives, as well as the potential positive impacts on student health, there is a need to understand the features and elements of F2S efforts that influence success.

**Figure 1:**
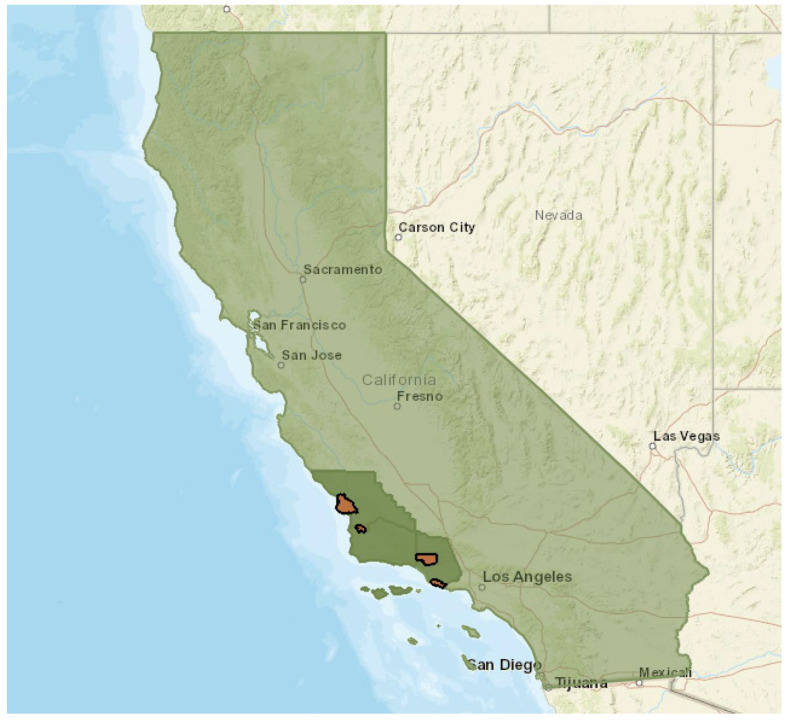
Location of the four farm-to-school systems (identified by orange shading) in three counties (identified by dark green shading) on the Central Coast of California (state identified by light green shading) included in the study.

This study applied a system mapping approach grounded in the Socio-Ecological Model (SEM) [22] to examine the structural and relational features that shape F2S partnerships. SEM emphasizes that behaviors and change are influenced by interactions across multiple levels of influence: individual, interpersonal, institutional, community, and broader policy systems [23]. Using the SEM framework, we explored how specific elements and stakeholder relationships within and across four F2S systems support or constrain implementation efforts across local contexts. We hypothesized that successful F2S systems share features that promote implementation and sustainability, despite contextual variation. This research can inform future strategies for developing local technical assistance, policies, and program scaling models to enhance student access to and consumption of healthy, local foods. Additionally, this research was conducted as part of a wider needs assessment aimed at understanding the community contexts that shape nutrition and healthy eating behaviors, focusing on low-income communities across Ventura, Santa Barbara, and San Luis Obispo Counties in California. This study employed a mixed-methods, iterative design to develop and compare system mapping diagrams across four F2S partnerships located on California’s Central Coast (see Figure 1), including the Ocean View Unified School District Farm-to-School Partnership and the Ojai Unified School District Farm-to-School Partnership (Ventura County), the Santa Maria Bonita School District Farm-to-School Partnership (Santa Barbara County), and the San Luis Coastal Unified School District (San Luis Obispo County).

## 2. Materials and Methods

To explore which elements of farm-to-school (F2S) systems support or constrain implementation efforts on the Central Coast of California, system mapping was utilized. System mapping is a “systems thinking” technique that visualizes the elements, relationships, and feedback loops that constitute the system’s functioning over time [24]. System mapping also allows researchers to describe a holistic overview of a partnership that transcends disciplinary boundaries to gain insights into the “underlying dynamics that drive” F2S partnerships [25]. For these reasons, system mapping can lead to the identification of leverage points, or places in the food-to-school partnership where a small change can impact overall system performance or success. For example, system mapping has been utilized to explore F2S relationships in Vermont to “critically examine the leverage points that may drive positive change within and across the system” [19] p. 133. In this research, we constructed system diagrams of four F2S partnerships on the Central Coast of California and analyzed the diagrams for shared and context-specific leverage points. The practical steps we followed drew on an iterative qualitative system mapping approach [25], which has been adapted in prior F2S work [19].

### 2.1. Case Selection

Purposive, two-stage sampling was utilized. In the first stage, eight F2S partnerships operating in San Luis Obispo, Santa Barbara, and Ventura Counties were identified through stakeholder referrals. In the second stage, four F2S systems that met all of the following inclusion criteria were retained: (1) active local procurement of California-grown product during the study year; (2) educational programming (e.g., garden lessons or farm field trips) linked to the partnership; (3) partner coordination evidenced by routine communication between schools and at least one external partner; (4) leadership-model (i.e., district-led or community-organization-led structures); and (5) feasibility of in-person engagement within the researchers’ Central-Coast assignment areas. All F2S systems were located on California’s Central Coast and represented differing levels of institutionalization, funding support, and partner engagement.

### 2.2. Data Collection

Data collection occurred through three interrelated phases: (1) review of public information and published research, (2) in-person site observations, and (3) local expert resources. Data were gathered in spring and summer 2025 using three complementary sources. (1) Public information and published research (approximately 6–10 per system) including school wellness policies, grant reports, public news reports, board minutes, and partner websites, were reviewed to identify system partners, activities, and contextual factors. This information provided a foundational understanding of each F2S system. (2) The researchers also conducted site visits for each F2S system, ranging from 3–5 h per system, where semi-structured field notes were captured to document (a) key system activities/interactions, (b) presence of partner organizations and elements, (c) physical food environments (menus, signage, storage, delivery areas), and (d) observational insights focused on how partnerships functioned in practice, including alignment or divergence from public data source accounts. Photographs were taken, with permission, to further contextualize the site findings. (3) Local experts, including food service directors, farmers, food hub staff, nonprofit coordinators, and Cooperative Extension personnel, provided resources that filled data gaps and contextualized findings. These resources were shared through informal, unrecorded conversations, e-mail exchanges, and the sharing of internal documents. These data collection methods supported the development of visual system diagrams, which were subsequently analyzed to identify common structural elements and context-specific adaptations across locations.

### 2.3. System Mapping and Validation

The researchers synthesized data from all three sources to create a contextual summary and system map for each F2S partnership, aligning with the iterative, qualitative system mapping protocol utilized in this study [25]. The researchers met regularly to discuss and interpret data, as well as to identify additional areas of inquiry. Two researchers independently coded data to identify system elements and links, then reconciled discrepancies through discussion. Diagrams were drafted in Canva with a standardized icon library; the first researcher built the maps, and the other performed a line-by-line review. Similarly, one researcher developed descriptive, contextual summaries for each partnership, which were then reviewed by the first researcher. Diagrams and contextual summaries were member-checked by 2–4 local experts (total *n* = 10) via e-mail or a virtual meeting. Revisions were incorporated iteratively, and key pre- and post-feedback versions were archived. Reliability was supported through dual review and triangulation across documents, observations, and expert insights.

### 2.4. Comparative Analysis

Comparative analysis of the system maps was conducted to identify both shared and divergent features utilizing a three-step process [26]. The first step in the analysis identified the presence and number of core elements and partners, as well as those that are absent, across the system maps. In the second step, the pathways and relationships between elements and partners were examined for (1) degree—the number of pathways connected to specific elements; (2) betweenness—the elements that are “between” pathways, indicative of elements that may be bridges or blocks to system function; and (3) centralization—relationships of elements in the system map to identify elements that are central to the system. In the third step, we examined the presence and absence of features for each element. Patterns were synthesized narratively; no statistical network software was applied, consistent with the exploratory scope of the study.

### 2.5. Rigor

Although this was an exploratory study, credibility was strengthened through triangulation of three separate data sources, as well as member-checking. The researchers sought dependability and confirmability through reconciliation discussions, as well as archiving diagram versions.

## 3. Results

### 3.1. Santa Maria Bonita School District Farm-to-School Partnership

Located in northern Santa Barbara County, the Santa Maria Bonita School District (SMBSD) F2S Partnership supports 21 school sites, serving approximately 17,165 students in grades K-8, within the community of Santa Maria [27]. The community’s Mediterranean climate, soils, and proximity to the Pacific Ocean create long growing seasons and support a strong agricultural industry. The community is surrounded by approximately 40,000 acres of row crops, including “strawberries, wine grapes, celery, lettuce, peas, squash, cauliflower, broccoli, spinach, and beans” [28].

This school district serves more than 25% of the students enrolled in public schools in the county [27]. In the school district, the majority of students (95.3%) are Hispanic/Latino [29] and most (56.5%) are English-language learners [30]. The majority of students (92.3%) are identified as living in socioeconomically disadvantaged circumstances [30]. Student academic outcomes are below “standard” in all reported areas of academic performance, and 12.5% of students in the district are chronically absent from school [30]. All students in the Santa Maria Bonita School District are eligible to receive free school meals through the Universal School Meals program and during the summer [31].

**Figure 2:**
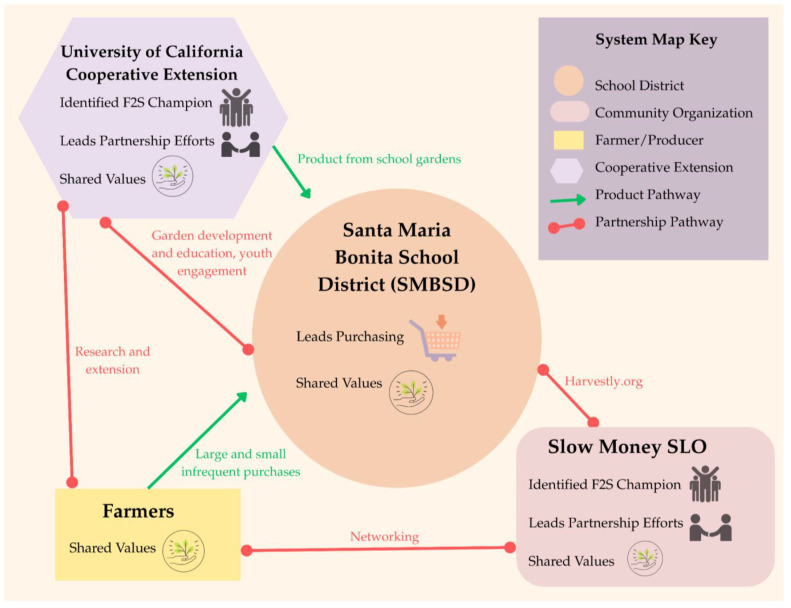
System map of the Santa Maria Bonita School District Farm-to-School Partnership.

While there are a number of regional networks, foundations, and community groups that support F2S efforts throughout the surrounding city and county, the researchers were unable to identify any records that the Santa Maria Bonita School District F2S Partnership is supported through these efforts. Similarly, most farmers’ markets in neighboring communities within the county accept EBT and/or provide Market Match benefits for qualifying low-income shoppers; however, the only farmers’ market located within the Santa Maria Bonita School District does not [32]. As a result, families within the district have reduced purchasing power at this market when compared to others in neighboring communities. Within this wider context, the researchers mapped the Santa Maria Bonita School District F2S Partnership (see Figure 2).

In their 2022–2023 SMBSD Triennial Wellness Assessment for the Santa Maria Bonita School District (SMBSD) Wellness Policy and Implementation Efforts [33], the district noted that they worked on a farm-to-school “initiative focusing on direct procurement of fresh fruits and vegetables from local farmers and educational farmers’ markets [at] school sites” during the review period. However, on their WellSAT 3.0 [34] (a Wellness School Assessment Tool created by the University of Connecticut), the district ranked itself as 1 out of 2 on “nutrition education addresses agriculture and the food system.” This assessment result is validated by research that indicates that the Santa Maria Bonita School District partners with the University of California Cooperative Extension in Santa Barbara County to develop and maintain school gardens, as well as garden-based nutrition education through the CalFresh Healthy Living, UC programming at four of the twenty-one school sites. This collaboration includes an annual Cooking Academy [35] hosted for students in the school district, as well as weekly leadership programs that engage students in teaching their peers and families about healthy and local foods [36].

On the WellSAT 3.0 (Wellness School Assessment Tool), the district ranked itself as 0 out of 2 on “addresses purchasing local foods for the school meals program.” While this score indicates the school does not actively purchase local foods for the school meal program, research found that the district does procure a limited amount of local foods through Harvestly [37]. This is a nonprofit program that operates a procurement platform, which includes the Santa Maria Bonita School District and local food producers as participants in the regional F2S efforts [38]. The nonprofit hosted local F2S Mixers—networking events that allowed farmers and representatives from the school district to meet [38]. Araceli Gaspar of Sunlife Farm a local strawberry farm in Santa Maria, California, operates approximately 100 acres of farmland annually [39] and participates in regional F2S efforts [38]. She described her motivation for delivering two-pallet strawberry orders to the district’s central receiving facility on an infrequent basis over the past three years: “We know where our product is going. Knowing that it’s going back to the community, because it’s here locally, gives a more rewarding feeling than selling it to a shipper. Then, we honestly don’t know who it goes to or who our consumers are. So, that’s really what drew our attention. I’ve also navigated to see if I could work with local food banks, but they get smaller-scale growers, I would want to work with them as well, because we know where our fruit is going” [40]. The desire to know the consumer, along with minimal logistical barriers and a user-friendly procurement platform, motivates Gaspar to continue to supply strawberries to the school district despite infrequent purchases of varied quantities.

### 3.2. San Luis Coastal Unified School District Farm-to-School Partnership

Located along the central coast of San Luis Obispo County, the San Luis Coastal Unified School District (SLCUSD) F2S Partnership supports 15 school sites, serving approximately 7817 students in grades K-12 [41], within the communities of San Luis Obispo, Edna Valley, Pismo Beach, Avila Beach, Los Osos, and Morro Bay. The coastal communities spanning the district areas are characterized by a Mediterranean climate with cool marine influences, fertile soils, and a long growing season. These conditions support a robust agricultural economy, with top crops including wine grapes, strawberries, cattle, broccoli, and avocados [42].

This school district serves 23.8%, or nearly one-quarter, of the students enrolled in public schools in the county [41]. In the school district, the majority of students (56.2%) are White and/or (30.3%) Hispanic/Latino [41]. Less than a tenth (9.1%) are English-language learners [43]. Less than half of students (43.5%) are identified as living in socioeconomically disadvantaged circumstances [43]. Student academic outcomes are above “standard” in all reported areas of academic performance, and 15.4% of students in the district are chronically absent from school [43]. All students in the San Luis Coastal Unified School District are eligible to receive school meals year-round through the Universal School Meals program, which includes foods purchased from local producers [44] and student access to free meals during summer months [45].

**Figure 3:**
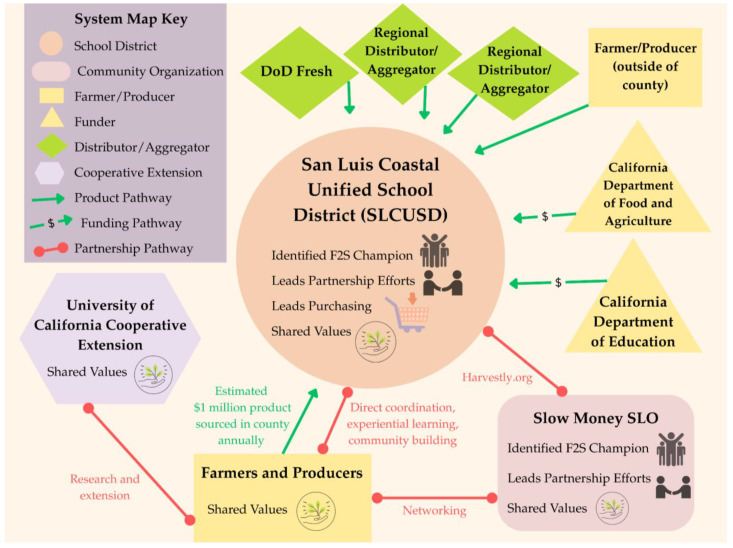
System map of the San Luis Coastal Unified School District Farm-to-School Partnership.

There is strong support in the community for ensuring young people have access to nutritious, local foods. In the area, there are numerous regional networks, foundations, and community groups that support F2S efforts in the community. Most farmers’ markets in the communities within the county accept EBT and/or provide Market Match benefits for qualifying low-income shoppers [46]. As a result, families within the district have increased purchasing power at the markets in their communities. Within this wider context, the researchers mapped the San Luis Coastal Unified District F2S Partnership (see Figure 3).

The San Luis Coastal Unified School District (SLCUSD), under the leadership of Erin Primer, Director of Food and Nutrition Services, has undergone a multi-year transformation in its school meal program, becoming “the first school district in California to achieve Eat Real Certification at the Silver Level” [47]. The district shifted from a heat-and-serve model to a system rooted in scratch cooking, local food procurement, organizational values alignment, student ‘taste tests,’ and local assessments [48,49,50,51]. Through sustained investments in infrastructure, staff training, and community partnerships, the district now operates a large-scale operation that feeds approximately 8500 students daily (~50% student participation rate) while simultaneously advancing food literacy and student engagement with the local food system [44,48,49,50,51]. These changes are supported by the district Wellness Advisory Council, including school personnel, parents, and community members, which developed school policy: “The San Luis Coastal Unified Food Services will attempt to coordinate its menus with seasonal production of local farms and with production in school gardens in order to reflect seasonality and local agriculture” [52] page C.

The SLCUSD F2S Partnership has developed into a comprehensive program centered on local procurement, cultural transformation, and educational engagement. In the 2022–2023 school year, the district spent nearly USD 1 million with 20 local farms and 25 food businesses, guided by Food Service Director Primer’s “start first with local” philosophy [53]. Her approach balances flexibility and relationship-building through diverse procurement methods, including micro-purchasing and adaptive menus, and is supported by the Harvestly procurement platform [37] and programs like the USDA DoD Fresh Fruit and Vegetable Program [54]. SLCUSD fosters deep partnerships with local producers, such as Kandarian Organic Farms for regenerative grains, Hearst Ranch for meat, Edna Bakery for bread items, and multiple local strawberry growers. During a site visit, the researchers observed that sacks of red lentils and quinoa from Kandarian Organic Farms were visible in pantry storage, serving as evidence of how these products contribute to the district’s scratch-cooking practices. The partnership supports local farms as well as creating meaningful student connections to local food systems and nutrition through events like farm visits [55], on-site education, and school-based learning, like summer culinary classes [56]. Similarly, the researchers observed that locally sourced items were accompanied by informative signage in the cafeteria, sharing photos and stories of the farmers and producers behind the ingredients to build interest and connection with students during school meals.

Strategic grants have supported the growth of the F2S partnership, but the district remains committed beyond funding. To reinforce skills and culture, the district invests in culinary training and leadership development programs for SLCUSD personnel, even supporting staff participation in multi-day workshops at the Culinary Institute of America. Primer explained that working in food service in SLCUSD is “a real culinary pathway,” emphasizing that they hire “people who are connected, who want to do scratch cooking, and who see the worth in that work” [53]. The SLCUSD food service employment model offers full-time positions, culinary training, a mission-driven environment, as well as a Culinary Food Service program through their adult school to prepare community members with the training needed to succeed in work environments like the SLCUSD food service [57].

### 3.3. Ocean View School District Farm-to-School Partnership

Located on the central coast of Ventura County, the Ocean View School District (OVSD) encompasses four schools serving approximately 2077 students in grades K-8 [58], in the city of Oxnard. The coastal communities spanning the district areas are characterized by a Mediterranean climate with cool marine influences, fertile soils, and a long growing season. These conditions support a robust agricultural economy, with major economic crops including strawberries, celery, tomatoes, and lettuce [59].

This school district serves less than 2% of the students enrolled in public schools in the county [58]. In the school district, the majority of students (87.4%) are Hispanic/Latino [58]. Over half of the students (55.7%) are English-language learners [60]. Over three-quarters of the student population (77.7%) are identified as living in socioeconomically disadvantaged circumstances [58]. Student academic outcomes are below “standard” in all reported areas of academic performance, and 19.5% of students in the district are chronically absent from school [60]. All students in the Ocean View School District are eligible to receive school meals year-round through the Universal School Meals program, which includes foods purchased from local producers [61], and students can also receive free meals during the summer [61,62].

**Figure 4:**
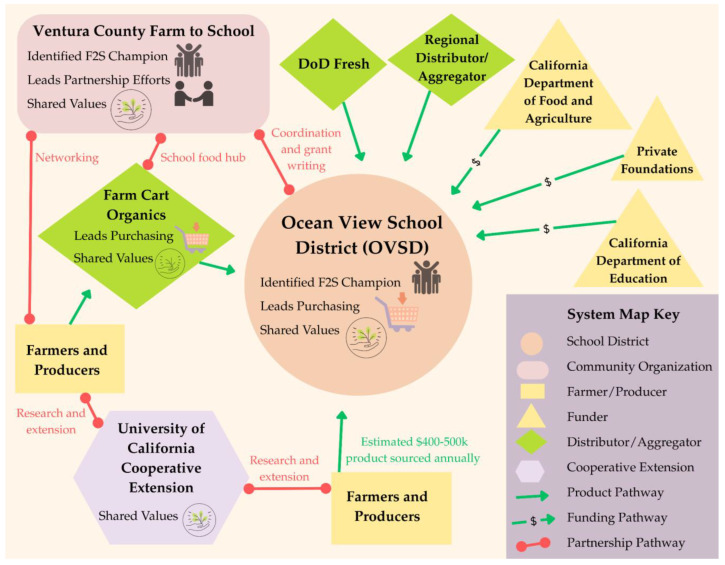
System map of the Ocean View School District Farm-to-School Partnership.

There are several F2S partnerships in the city of Oxnard ensuring nutritious, local foods are accessible through school meals [63,64,65]. There are also numerous regional networks, foundations, and community groups that support F2S efforts in the city. At the same time, while some farmers’ markets within Ventura County accept EBT and/or provide Market Match benefits for qualifying low-income shoppers, the farmers’ market in the city of Oxnard does not allow shoppers to use food benefits [66]. As a result, families within the district have decreased purchasing power at the farmers’ market in their community. Within this wider context, the researchers mapped the Ocean View Unified School District F2S Partnership (see Figure 4).

Under the leadership of Nutrition Services Director Allison England, the Ocean View School District has developed a mission-driven meal program, which has been recognized as a leader in “healthy school pathways” through the implementation of “best practices in scratch-cooked school food, local food systems, and sustainable practices” [67]. As all five schools in the district are served by a single central receiving bay, the district has been able to operationalize values-based procurement, prioritizing local sourcing, organic products, and compostable packaging [68]. The F2S partnership has strong internal support from district leadership and strategically utilizes intermediaries between farmers and the school district to source an estimated 40–50% of its food locally, with an annual purchasing budget of USD 400,000–500,000 [69]. Farm Cart Organics, a distributor/aggregator that consolidates deliveries from multiple farms for schools, has been instrumental in bridging the gap between small and mid-size farm operators and the school district. By consolidating these small orders and meeting liability insurance requirements, Farm Cart enables districts such as OVSD to receive products from multiple farmers in one delivery. During site observation, the researchers observed a delivery of a small quantity of tree fruits in a small pickup truck. A food purchase of this size would not be feasible for the district and is made possible by Farm Cart, which purchases products from multiple small growers [70]. Meanwhile, the Ventura County Farm to School, a local nonprofit, relieves many administrative burdens small school districts typically face, such as supporting the district with product sourcing, grant writing, and coordination of procurement logistics [71].

The success of this relationship is clear through the impact of the grant and foundation support obtained. For example, CDFA grant funds awarded to Ventura County’s Farm to School program [72] enable the Ocean View School District to purchase local, regenerative, and organic produce that would otherwise be financially infeasible to purchase [70]. Other grant support, like the California Department of Education Kitchen Infrastructure and Training (KIT) Fund, has enabled the district to engage food service personnel in training, field trips, and extended hours, while also supporting infrastructure upgrades [73,74]. A Farm to School Incubator Grant from CDFA provided funding to “improve student nutrition knowledge, increase consumption of fruits and vegetables, enhance appreciation for local food systems, and strengthen partnerships within the community” through expanded experiential learning for students [75].

All these efforts have contributed to enhanced food quality and improved employee satisfaction. “There’s pride now in what we’re cooking,” Food Service Director England explained, as school meals feature culturally relevant and locally sourced ingredients [69]. This commitment to values-based cooking was evident during a site observation, when the food services team prepared a Mexican-style shrimp cocktail for Cinco de Mayo, featuring local shrimp from Get Hooked Seafood along with locally sourced ingredients like lemon slices and fresh avocado. The dish not only showcased high-quality local products in school food but also reflected the district’s commitment to offering culturally relevant meals that resonate with their students.

### 3.4. Ojai Unified School District Farm-to-School Partnership

Located in a small valley in Ventura County, the Ojai Unified School District (OUSD) F2S Partnership supports seven school sites, serving approximately 2008 students in grades TK-12 [76], within the communities of the Ojai Valley. The valley encompassing the district is characterized by a Mediterranean climate with greater temperature variations due to distance from the ocean and surrounding mountain ranges, fertile soils, and a long frost-free growing period. These conditions support a niche and productive agricultural economy, with major economic crops including citrus, avocado, and olives for oil production [59].

This school district serves less than 2% of the students enrolled in public schools in the county [76]. In the school district, the majority of students (53.8%) are White and a significant portion of students (38.9%) are Hispanic/Latino [76]. Less than a tenth of the students (8.7%) are English-language learners [77]. Fewer than half of the student population (40.3%) is identified as living in socioeconomically disadvantaged circumstances [76]. Student academic outcomes are below “standard” in all reported areas of academic performance and 19.2% of students in the district are chronically absent from school [77]. All students in the Ojai Unified School District are eligible to receive school meals year-round through the Universal School Meals program, and students can also receive free meals during summer [62].

**Figure 5:**
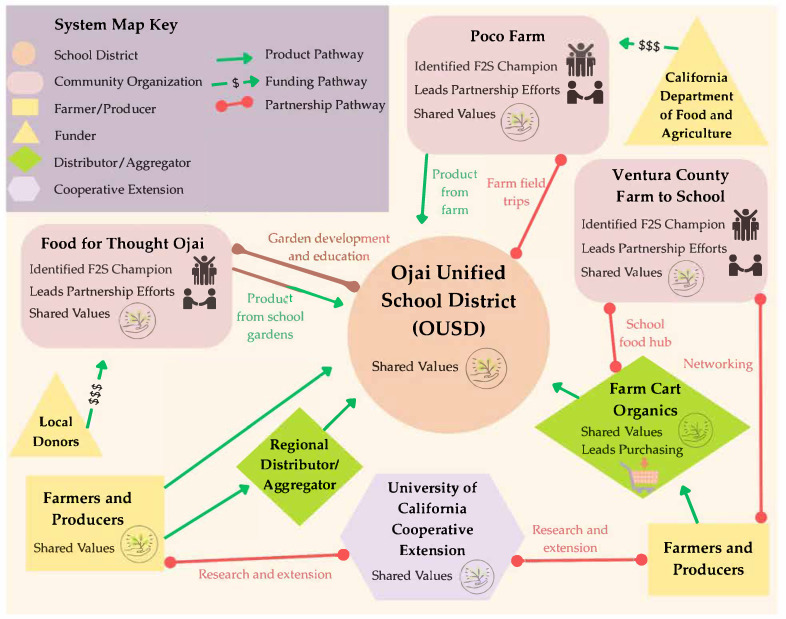
System map of the Ojai Unified School District Farm-to-School Partnership.

In the Ojai Valley, the inclusion of F2S activities is common at the numerous private schools (including at Monica Ros Elementary School, The Thacher School, Ojai Valley School, and Oak Grove School), as well as through the Ojai Unified School District F2S Partnership, ensuring nutritious, local foods are accessible to students in the community through school meals and activities [78,79,80,81,82]. There are also numerous regional networks, foundations, and community groups that support F2S efforts in the Ojai Valley [83,84,85,86]. Some farmers’ markets within Ventura County accept EBT and/or provide Market Match benefits for qualifying low-income shoppers, including the farmers’ market in the Ojai Valley, which allows shoppers to use food benefits to shop but does not provide Market Match [66]. Within this wider context, the researchers mapped the Ojai Unified School District F2S Partnership (see Figure 5).

The initial F2S partnership in the Ojai Unified School District (OUSD) was spearheaded by Food for Thought Ojai, a nonprofit organization founded in 2002. This collaboration aimed to enhance student health and academic performance by integrating fresh, locally sourced produce into school meals and providing nutrition education [83]. Their work aligns with California State Academic Standards while emphasizing: “(1) nutrition education, (2) garden-based learning, (3) agricultural literacy through farm field trips, and (4) advocating for fresh, local, seasonal produce in all school meals” [85]. Food for Thought Ojai partners with OUSD’s Nutrition Services Director, a long-standing network of local growers and nonprofit partners like Ventura County Farm to School [87] to ensure that students receive fresh, local food through the OUSD F2S Partnership. The *Harvest of the Month* campaign has been implemented across the district, integrating lessons into the cafeteria and classrooms [83]. School gardens and garden-based education are led by Food for Thought Ojai at all OUSD elementary schools [85]. The district has created a pathway for student-grown produce from the school garden to be utilized in the school cafeteria [83,85].

Despite OUSD’s small size, coordination of funding, procurement, and relationships is complex. The district works with distributors/aggregators such as Farm Cart Organics to streamline ordering, delivery, and invoicing for small to mid-sized farms [88]. Costs remain a key challenge, and the partnership utilizes multiple funding sources, including federal and state grants, foundation support, and community fundraising, to sustain local procurement throughout the school year [83]. For example, the OUSD F2S Partnership is supported by CDFA Farm to School grant funding to provide field trips for all students to Poco Farm, a 4-acre nonprofit regenerative learning farm [89]. Additionally, these funds support Poco Farm in collaborating with OUSD Food Service to adapt production to meet the specific needs of the district for school meals [89].

Vital to the partnership’s success is a strong values alignment across stakeholders. The system map consistently reflects the partnership’s commitment to the health of the Ojai Valley’s youth, community, and environment. The OUSD Food Service states, “We are nourishing the heart and future of the Ojai community with healthy school meals made from whole, local, and organic ingredients” [81]. Similarly, Farm Cart Organics explains: “We have partnered with farmers who share the same dedication to creating a healthier, happier planet and community by supporting biodiversity, workers rights, and regenerative practices with every seed they sow” [88]. Poco Farms states that “our farm’s purpose is to cultivate in our community members and ourselves the compassion, connection and skill sets needed to thrive on our changing planet” [89]. As Nate Yeager, Executive Director, Food for Thought Ojai, reflected, “At a high level, everybody shares the same goal. Everybody wants to see better nutrition in school meals, less processed food, and more whole, local foods. Everybody wins, because you support our local farmers, our local growers, and you are directly supporting student health” [90]. The partnership’s success reflects the strong alignment of partners’ values across the system. However, the district’s Triennial Assessment and WellSAT 3.0 Scorecard suggest that some within the district see room for expansion of these efforts, scoring itself at a 1 out of 2 on the measure: “Nutrition education addresses agriculture and the food system;” and a 0 out of 2 on the measure: “Addresses purchasing local foods for the school meals program” [91].

### 3.5. Comparative Analysis of Farm-to-School Partnership System Maps

In accordance with the study methods, the first step in the comparative analysis focused on identifying the presence and number of core elements (or F2S partners) for each system, as well as those that are absent, across the system maps. The results of this analysis are included in Table 1. All partnerships have one or more of the following elements: school district, community organization, farmer/producer, and Cooperative Extension partners. Due to the nature of the study, each system included one school district. The number of community organizations present in each partnership was either one or three. Across all systems, farmers/producers were grouped by purchasing and distributing relationships, so these numbers do not reflect the count of participating businesses. The SMBSD F2S Partnership was the sole system where farmers/producers only sold and distributed to the district directly. In all other systems, farmers/producers may sell and distribute to the district or through a distributor/aggregator. The known number of distributors/aggregators in each system ranged from zero (SMBSD F2S Partnership) to three. Collectively, distributor/aggregator was the element with the greatest number of instances. Similarly, the number of known external funders in each system ranged from zero to three. Finally, Cooperative Extension was present in all systems, providing extension education and/or research to school personnel and students and/or farmers/producers.

In the second step of the comparative analysis, we examined the pathways between elements (or relationships between system partners). To determine the degree, or the number, of pathways connected to specific elements, we first analyzed the number of partnership pathways connecting to each element. The results of this analysis are in Table 2. The number of partnership pathways connected to the school districts ranged from one to two, with school districts most frequently connecting to community organizations and farmers/producers. Community organizations consistently had the greatest number of partnership pathways across the systems (ranging from two to four). The number of partnership pathways connected to farmers/producers was either two or three, and often connected to Cooperative Extension or community organizations. There were no pathways for distributors/aggregators in two systems and only one pathway in each of the remaining two systems, connecting to community organizations despite representing the element with the greatest frequency across all systems. Funders had no partnership pathways present across all systems. Finally, Cooperative Extension had one partnership pathway in the SLCUSD F2S Partnership and two partnership pathways in each of the remaining systems, most frequently connecting to farmers/producers.

Next, we analyzed the number of product/grant distribution movement pathways connecting to each element, representing the disbursement of food or grant funds to support F2S activities within the partnership. The results of this analysis are shown in Table 3. The number of product/grant distribution pathways connecting to school districts ranged from two to seven across the systems, making the school district the element with the greatest number of product/grant distribution pathways across all systems. In most systems, there were zero product/grant distribution pathways connected to community organizations; however, the OUSD F2S Partnership was the exception, with four product/grant distribution pathways connected to community organizations. For the farmer/producer element, the product/grant distribution ranged from one (SMBSD F2S Partnership) to two in the remaining systems.

There were three to four product/grant distribution pathways connected to the distributor/aggregator elements in three partnerships; however, the SMBSD F2S Partnership had zero. Similarly, there were two to three product/grant distribution pathways connected to the funder element in three of the systems, with zero in the SMBSD F2S Partnership. Finally, Cooperative Extension was connected to one product/grant distribution pathway across the systems, appearing only in the SMBSD F2S Partnership.

The next aspect of the analysis examined whether there were elements that were in “between” pathways in the systems. Betweenness seeks to identify elements that may be bridges or blocks in the systems’ functions. The results are shown in Table 4. The school district and farmer/producer elements did not appear to occupy a “between” pathway position in any of the systems. Community organizations and the distributor/aggregator element were identified as between-pathway elements in two different systems, with both elements occupying between-pathway positions in two different locations in the OUSD F2S Partnership. The funder and Cooperative Extension elements each appeared in a between-pathway position in a separate single system. Collectively, there were zero to four elements occupying between-pathway positions across all systems, with two occurrences appearing in half of the systems.

The last consideration for the second stage of the comparative analysis identified the central element(s)—or the partner the system was centered around—across the system maps. Due to the nature of the study, it was expected that the school district would appear in the central position of each system, which was verified. However, the researchers noted that in the SMBSD F2S Partnership, there was a second element that also occupied a similar centralized position in the partnership, namely Cooperative Extension.

In the final step of the comparative analysis, the researchers examined the presence and absence of features for each element across the system maps. The results are shown in Table 5. Across all systems, the feature that appears with the greatest frequency (*n* = 17) is shared values, spanning all elements of the systems except the funder. Three elements received the “champion” feature across all systems, including school district, community organization, and Cooperative Extension. In most systems, the element assigned the “leads purchasing” feature was the school district; however, the distributor/aggregator element also received the feature in two systems. Three elements received the “leads partnership” feature across all systems, including school district, community organization, and Cooperative Extension. Funder was the only element that was not assigned any feature across all systems. In contrast, the element that received the greatest number of features across all systems was community organization, receiving 12 feature applications, including “champion” (*n* = 4), “shared values” (*n* = 4), and “leads partnership” (*n* = 4). The only feature that was not assigned to community organizations across all systems was “leads purchasing.” Across all systems, school districts had each feature applied at least once, with “shared values” occurring in all systems. For the element farmer/producer, “shared values” was the only feature applied and only in three systems (*n* = 3).

## 4. Discussion

Farm-to-school (F2S) partnerships operate at the intersection of food systems, education, and public health, requiring coordination across diverse sectors. The comparative analysis of the four F2S system maps included in this study identified several characteristics of successful F2S systems on the Central Coast of California, as well as supportive features and system challenges. In this study, “success” was defined not by a single outcome, but by a collection of features that support implementation and sustainability across diverse contexts. Drawing from the Socio-Ecological Model, as applied to early childhood food environments [92], success was conceptualized as the presence of key structural and relational elements, such as stakeholder alignment, identified champions, institutional support, and multi-directional communication and distribution pathways, that collectively enable effective F2S partnerships. Rather than focusing solely on procurement volume or educational outcomes, we examined how these systemic features contribute to a school food environment that leads to student access to and consumption of healthy, local foods.

Utilizing this information, the researchers considered whether there are universal characteristics across all partnerships. Shared values across system partners is a significant characteristic in all F2S systems included in this research. As University of California Cooperative Extension Research Advisor Tuohey-Mote indicated, “farmers want to feed people, and so do school districts,” highlighting an alignment of mission across sectors [93]. For many farmers, providing food to children feels meaningful. “They really like the idea of their food being fed to the community, and particularly to children” [94]. While motivations and values may differ among system partners, there is a perception within the systems that most partners have shared values that enhance the partnership. In different cases, the value may range from supporting child nutrition and health, to valuing local agriculture and local food systems, to supporting sustainable interventions for improved environmental outcomes. Prior research examining a range of inquiries involved in F2S efforts also describes that stakeholder groups in F2S systems tend to share values that support the success of the systems [95,96,97,98].

Similarly, each system has one or more identified champions who support multiple functions in the partnership and in most cases also initiate or lead the partnership activities. In F2S partnerships, champions often “play a pivotal role in linking other stakeholders and maintaining the energy, enthusiasm and forward momentum of local [F2S] efforts” [99] p. 111. Prior research has found that champions can be found across the range of partners involved in F2S systems, including individuals working with partners along the supply chain, schools, community organizations, and government [100,101]. University of California Cooperative Extension Advisor Ben Faber described champions as essential to F2S progress, explaining that organizations have key individuals who often seem to “run the whole thing,” and warning that “without these leaders, progress stalls” in the F2S partnership [102]. At the same time, an over-reliance on a singular champion may also be identified as a weakness for system sustainability over time. Champions in the included systems were engaged in a wide range of responsibilities, from grant writing and professional development for personnel to direct engagement with students and families, demonstrating their adaptability and critical importance in sustaining F2S partnerships. Over-reliance on champions, particularly those in volunteer or community roles, may lead to system collapse or slowdown if an existing champion withdraws because of burnout, turnover, or shifting personal commitments. To prevent this potential, systems should ensure that formal structures and succession planning are in place to distribute champions’ knowledge, relationships, and decision-making authority.

Across the systems, supportive features were also identified. While the SLCUSD F2S Partnership demonstrates that having partners who support the distribution of food and funds to school districts is not required for success, these partners do play key roles in the smaller partnerships. Similarly, the number of product/grant distribution pathways connected to the school district, and in some cases the community organization(s), may also be a supportive factor and is a representation of the system’s capacity for and/or volume of food handled and grant funds secured to support student access to local foods. This aligns with prior research findings that districts were able to acquire the greatest amount of local foods when they utilized marketing channels, including direct procurement, distributors/aggregators, food purchasing co-ops, and government procurement programs [103]. The number of partnership pathways also seems to increase with the expansion of the F2S partnership. While the increase in the number of partnerships can increase the complexity of the management of the partnership activities, it appears that community organizations may manage more partnership pathways as the partnership grows, which can reduce the burden for school districts and farmers/producers. These findings underscore the relevance of the Socio-Ecological Model in understanding F2S systems, as partnerships that demonstrated alignment across policy, organizational, and interpersonal levels may be better positioned to sustain and expand F2S implementation.

The findings also have insights for addressing system challenges. While there has been success in obtaining external funds in some systems, the analysis highlighted the lack of partnership pathways between funders and other partners in the system, in particular the lack of partnership pathways between funders and the students consuming local foods. Similarly, the funders were the only partners in the partnership who were not clearly identified as having shared values across all systems. There may be an opportunity to increase the impact of the relationships with the external funders to cultivate a greater sense of shared values and championing for the partnership through activities identified in the data collection phase, such as inviting funders to visit sites, participate in educational programs, and shape future F2S programming directions. Research indicates that donors value partners and programs that build trust and transparency through relationship-building when engaged in financial giving [104]. These efforts could create pathways between funders and the school districts, as well as cultivate “shared values” and potentially new “champions” for the partnership, which may increase the longevity of the funding relationships that support the partnership.

Two school districts included in the analysis indicated room for improvement in F2S efforts based on recent self-assessments. In both districts, the partnerships were led by community organizations or Cooperative Extension rather than the school district. This finding suggests that F2S partnerships can be successful in school districts where the school is unable or unwilling to champion and/or lead the partnership. While this research did not assess school districts’ interest, prior research highlights how contextual factors such as poverty rate, educational attainment, and median household income play a significant role in a district’s ability to initiate F2S programming, even when interest is present [105] p. 338. For school districts with an interest in initiating F2S partnerships, additional barriers such as inadequate kitchen facilities, food safety requirements, and limited food preparation capacity among school food service staff can constrain implementation [5]. Power dynamics and issues of equity emerged across the systems, particularly in underserved districts. In the Santa Maria Bonita School District, Cooperative Extension served as the primary driver of F2S implementation. While this external leadership helped enable the program’s launch, the school district’s limited internal capacity resulted in a more passive role in shaping or sustaining the partnership, highlighting how power over programming and priorities may reside outside the institution most directly serving students. As funding for Cooperative Extension’s nutrition education program in the community faces federal elimination in 2025, students in this community may no longer have access to the F2S programming that their peers will continue to receive where Cooperative Extension is not the primary driver. Similarly, in the Ojai Unified School District, key F2S initiatives, including garden-based education and farm field trips, were led by nonprofit partners rather than the district itself. Despite this active programming, the district reported low scores on relevant WellSAT 3.0 self-assessment items [91], suggesting a disconnect between implementation and institutional ownership. These examples illustrate how resource constraints and reliance on external actors can influence power dynamics and long-term sustainability, particularly in under-resourced districts. In this study, the two districts identified as school-based champions received grant funding to support facility improvements and professional development. Their leadership was reflected in the greater number of product and fund movement pathways centered around the school district. This capacity was reflected in the structure of their system maps, suggesting that districts can serve as effective leaders of F2S efforts. However, in other cases, there may be a disconnect between F2S practices and how districts perceive and report their involvement, as noted by the Ojai Unified School District. This discrepancy illustrates how self-assessments and written wellness policies may not always capture the full extent of partnership-driven activities, particularly when those initiatives are supported by external organizations. While stronger wellness policies are generally associated with implementation [106], other research suggests that written policy quality does not consistently correlate with observed school nutrition environments [107]. These inconsistencies highlight the need to complement policy and self-assessment reviews with direct observation and stakeholder input to more accurately assess implementation and guide strategies for improvement.

Across all F2S partnerships included in this study, Cooperative Extension was identified as a contributing partner on each system map. In these partnerships, Cooperative Extension contributed to the development and maintenance of school gardens, student nutrition education, professional development (extension education) for farmers/producers, conducted related research, and supported stakeholder connections/collaborations. Across the four systems, Cooperative Extension advisors filled a range of roles, including educator, coordinator, relationship-builder, and champion. These contributions are consistent with national research in the United States that recognizes Cooperative Extension as a key connector and facilitator of F2S partnerships, serving as a catalyst of strategic linkages between stakeholder groups, developer of local food policy, and facilitator of garden-based and experiential learning [108,109,110]. The broad knowledge base, research and extension expertise, and longstanding community relationships make Cooperative Extension uniquely positioned to advance F2S efforts across diverse systems throughout the United States.

### 4.1. Limitations

There are several common research limitations associated with system mapping research, especially in interdisciplinary fields and complex programming environments like food systems, education, and public health. The data collection and system maps rely on public information and expert review, which can reflect personal or organizational perspectives rather than objective system realities. Similarly, researchers’ subjectivity and bias may influence definitions of system boundaries and depth, which in turn affect the data included, analysis, and interpretation. As the systems are constantly evolving, they provide a snapshot reflecting a single point in time, which will become less reflective of the current system over time. The system maps are partial as not all actors and feedback loops are included. For example, this study did not include student or parent perspectives. The boundaries for the systems maps included in this study were limited to partners that directly impact the availability of local foods for consumption by students in the identified districts; however, the system is impacted by partnerships that extend outside this reach, including additional school districts receiving foods from the same producers and/or distributors/aggregators; regional and state networks that influence policy, systems, and environments; or federal decision-makers that determine funding priorities and manage F2S grants. In this research, the system mapping analysis is descriptive, so insights are exploratory and not definitive or causal. Finally, the findings of this research are specific to the four F2S partnerships mapped and have limited generalizability.

### 4.2. Practical Implications

The findings of this study have several practical implications. For individuals and organizations involved in existing F2S partnerships, this research highlights the role that a sense of shared values plays in the partnership. However, contributors’ values across the system may not be well known to all involved, so there may be an opportunity to engage partners in value-based programming or activities, such as creating a shared value statement for the partnership. The expanded partnerships and associated activities in some systems highlight the impact of successful fund development for F2S capacity (i.e., school kitchen renovation) and activities (i.e., farm field trips for students). This suggests that partnerships would benefit from fund development support, including champions who can draft grant proposals, as well as the opportunity to participate in grant writing programs. For school districts, organizations, and communities seeking ways to initiate or expand F2S partnerships, the shared features identified in this study can serve as a foundation for efforts in other regions of the United States. The role of Cooperative Extension varied across partnerships and showed the ability of Cooperative Extension to expand into existing F2S partnerships. Future Cooperative Extension efforts may focus on the development of school gardens, garden- and farm-based nutrition education for students and families, as well as technical assistance for farmers/producers (i.e., food safety considerations).

### 4.3. Research Implications

Future research on these F2S partnerships would benefit from in-depth research with partners and collaborators, including conducting formal interviews and survey research within the four systems to better understand perceived system gaps and challenges, as well as opportunities to expand partnerships. Collectively, the research on F2S initiatives would benefit from the development of validated tools to examine the impact of F2S partnerships on student nutrition and health outcomes. Researchers may also consider the value of system mapping F2S partnerships in other geographical areas and/or comparing system maps of F2S partnerships that received California Farm-to-School Incubator Grant Program funds with those that have not received these funds. Researchers who work in public health may consider how system mapping might inform additional programming that involves partnerships across different sectors.

## 5. Conclusions

This research utilized system mapping of four F2S partnerships on the Central Coast of California to identify components of F2S partnerships that support success and explore potential system challenges in order to guide the development of future strategies to support more adaptive and resilient F2S partnerships. This research found that shared values across system partners, having one or more identified champions working in the system, and partnering with Cooperative Extension are universal characteristics of effective F2S systems. Supportive features were also identified and included the system partners (such as distributors/aggregators or community organizations) that support the distribution of foods and funds to the school district, as well as the number of product/grant distribution pathways connected to school districts. Community organizations that manage more partnership pathways were seen in the larger partnership systems. System challenges were also identified. These included the lack of identified partnership pathways between funders and other system partners, which may contribute to a sense that funders may not share the same values as partners in the system or cannot be identified as partnership champions. In systems where school districts do not lead the partnership or serve as partnership champions, there may be a need for resources to support school district capacity to engage more deeply (i.e., professional training, kitchen renovations), as well as increased communication about program activities, impacts, and opportunities between the school districts and system partners. This research has provided insights to support the development of new resources that build on partnership strengths and support the navigation of critical system leverage points to expand F2S partnerships. Through the expansion and strengthening of these F2S partnerships, there is an opportunity to expand the systems’ ability to positively influence student health and nutrition behaviors through F2S procurement, school gardens, and student education.

## Figures and Tables

**Table 1:** Presence of core system elements across partnership system maps.

Element	SMBSD F2S	SLCUSD F2S	OVSD F2S	OUSD F2S
School District	1	1	1	1
Community Organization	1	1	1	3
Farmers/Producers	1	2	2	2
Distributor/Aggregator	0	3	3	2
Funder	0	2	3	2
Cooperative Extension	1	1	1	1
Total	4	10	11	11

**Table 2:** Degree of partnership pathways by element across systems.

Number of Partnership Pathways	SMBSD F2S	SLCUSD F2S	OVSD F2S	OUSD F2S
School District	2	2	1	3
Community Organization	2	2	3	3
Farmer/Producer	2	2	3	3
Distributor/Aggregator	0	0	1	1
Funder	0	0	0	0
Cooperative Extension	2	1	2	2
Total	8	7	10	12

**Table 3:** Degree of product/grant distribution pathways by element across systems.

Number of Product/Grant Distribution Pathways	SMBSD F2S	SLCUSD F2S	OVSD F2S	OUSD F2S	Total
School District	2	7	7	5	21
Community Organization	0	0	0	4	4
Farmer/Producer	1	2	2	2	7
Distributor/Aggregator	0	3	4	4	11
Funder	0	2	3	2	7
Cooperative Extension	1	0	0	0	1

**Table 4:** Identification of elements in positions “between” pathways across systems.

Element	SMBSD F2S	SLCUSD F2S	OVSD F2S	OUSD F2S
School District	0	0	0	0
Community Organization	1	0	0	2
Farmer/Producer	0	0	0	0
Distributor/Aggregator	0	0	1	2
Funder	0	0	1	0
Cooperative Extension	1	0	0	0
Total	2	0	2	4

**Table 5:** Identification of features present in each element across systems.

Element	Champion	Shared Values	Leads Purchasing	Leads Partnership
School District		SMBSD	SMBSD	
	SLCUSD	SLCUSD	SLCUSD	SLCUSD
	OVUSD	OVUSD	OVUSD	
		OUSD		
Community Organization	SMBSD	SMBSD		SMBSD
	SLCUSD	SLCUSD		SLCUSD
	OVUSD	OVUSD		OVUSD
	OUSD	OUSD		OUSD
Producer/Farmer		SMBSD		
		SLCUSD		
		OUSD		
Distributor/Aggregator		OVUSD	OVUSD	
		OUSD	OUSD	
Funder				
Cooperative Extension	SMBSD	SMBSD		SMBSD
		SLCUSD		
		OVUSD		
		OUSD		

## Data Availability

The original contributions presented in this study are included in the article. Further inquiries can be directed to the corresponding author.

## Abbreviations

The following abbreviations are used in this manuscript:

CDFA	California Department of Food and Agriculture
DoD	Department of Defense
F2S	Farm-to-school
KIT	Kitchen Infrastructure and Training
OUSD	Ojai Unified School District
OVSD	Ocean View School District
SLCUSD	San Luis Coastal Unified School District
SMBSD	Santa Maria Bonita School District
